# Phenotypic Screen of Early-Developing Larvae of the Blood Fluke, *Schistosoma mansoni*, using RNA Interference

**DOI:** 10.1371/journal.pntd.0000502

**Published:** 2009-08-11

**Authors:** Marina de Moraes Mourão, Nathalie Dinguirard, Glória R. Franco, Timothy P. Yoshino

**Affiliations:** 1 Departamento de Bioquímica e Imunologia, Instituto de Ciências Biológicas, Universidade Federal de Minas Gerais, Belo Horizonte, Minas Gerais, Brazil; 2 Department of Pathobiological Sciences, University of Wisconsin, Madison, Wisconsin, United States of America; McGill University, Canada

## Abstract

RNA interference (RNAi) represents the only method currently available for manipulating gene-specific expression in *Schistosoma* spp., although application of this technology as a functional genomic profiling tool has yet to be explored. In the present study 32 genes, including antioxidants, transcription factors, cell signaling molecules and metabolic enzymes, were selected to determine if gene knockdown by RNAi was associated with morphologically definable phenotypic changes in early intramolluscan larval development. Transcript selection was based on their high expression in *in vitro* cultured *S. mansoni* primary sporocysts and/or their potential involvement in developmental processes. Miracidia were allowed to transform to sporocysts in the presence of synthesized double-stranded RNAs (dsRNAs) and cultivated for 7 days, during which time developing larvae were closely observed for phenotypic changes including failure/delay in transformation, loss of motility, altered growth and death. Of the phenotypes evaluated, only one was consistently detected; namely a reduction in sporocyst size based on length measurements. The size-reducing phenotype was observed in 11 of the 33 (33%) dsRNA treatment groups, and of these 11 phenotype-associated genes (superoxide dismutase, Smad1, RHO2, Smad2, Cav2A, ring box, GST26, calcineurin B, Smad4, lactate dehydrogenase and EF1α), only 6 demonstrated a significant and consistent knockdown of specific transcript expression. Unexpectedly one phenotype-linked gene, superoxide dismutase (SOD), was highly induced (∼1600-fold) upon dsRNA exposure. Variation in dsRNA-mediated silencing effects also was evident in the group of sporocysts that lacked any definable phenotype. Out of 22 nonphenotype-expressing dsRNA treatments (myosin, PKCB, HEXBP, calcium channel, Sma2, RHO1, PKC receptor, DHHC, PepcK, calreticulin, calpain, Smeg, 14.3.3, K5, SPO1, SmZF1, fibrillarin, GST28, GPx, TPx1, TPx2 and TPx2/TPx1), 12 were assessed for the transcript levels. Of those, 6 genes exhibited consistent reductions in steady-state transcript levels, while expression level for the rest remained unchanged. Results demonstrate that the efficacy of dsRNA-treatment in producing consistent phenotypic changes and/or altered gene expression levels in *S. mansoni* sporocysts is highly dependent on the selected gene (or the specific dsRNA sequence used) and the timing of evaluation after treatment. Although RNAi holds great promise as a functional genomics tool for larval schistosomes, our finding of potential off-target or nonspecific effects of some dsRNA treatments and variable efficiencies in specific gene knockdown indicate a critical need for gene-specific testing and optimization as an essential part of experimental design, execution and data interpretation.

## Author Summary

RNA interference (RNAi) represents the only method currently available for manipulating gene-specific expression in human blood flukes, *Schistosoma* spp., although its application as a functional genomics tool in early intramolluscan larval stages has been limited to single gene analyses. Accelerating gene discovery efforts over the past 10 years have resulted in extensive, ever-increasing databases of genomic, transcriptomic and EST sequences. Unfortunately, our understanding of the function of the vast majority of these genes has not kept pace with their discovery, and this represents a significant barrier and the next real challenge for investigators of schistosomes, and other parasitic helminths. In the present study, we selected an array of 32 genes expressed in *S. mansoni* sporocysts to evaluate their susceptibility to double-stranded (ds)RNA treatment and to begin characterizing morphological phenotypes associated with a potential RNAi effect. Results demonstrate that gene knockdown and/or resulting phenotypes are highly transcript-dependent (specific dsRNA sequence used) and vary with time post-dsRNA exposure. Because of this potential variability in both transcript and phenotype expression in response to dsRNA treatment, our findings illustrate that, although a RNAi-type approach holds great promise as a functional reverse-genetics tool for larval schistosomes, its application requires caution in the design and execution of experiments and interpretation of results.

## Introduction

Digenetic trematodes (parasitic flatworms) of the genus *Schistosoma* infect more than 200 million people in over 70 developing countries [1], with an additional 770 million people worldwide at risk of becoming infected [2]. As causative agents of chronic, often severe morbidity and responsibility for an estimated 280,000 death per year in Africa alone [3], schistosomiasis ranks as one of the most important of neglected tropical diseases [4].

Although significant research effort and funding have been dedicated to the treatment and control of schistosomiasis, including sanitary measures, suppression of the snail intermediate host, and chemotherapeutic interventions, there has been little change in the overall disease prevalence [5]. Progress in vaccine development has been very slow, and although several antigens, some of which are currently under clinical trial, have shown limited promise in rodent and primate challenge experiments, prospects are not good for an effective, highly protective vaccine in the foreseeable future [6],[7]. Clearly there continues to be a pressing need for new strategies to break the cycle of schistosome transmission to the human population [8]–[10].

In view of the limited options available for controlling schistosomiasis in both the human host and snail vector, it is important that research focus on obtaining information that can be translated into new tools for parasite control. To that aim, genomic, transcriptomic and proteomic approaches offer strong possibilities to discover new potential targets for vaccines, develop new drug candidates, and provide a better understanding of basic molecular mechanisms underlying host-parasite interactions. The *S. mansoni* Genome Project and data generated by various gene discovery efforts using expressed sequence tags (ESTs) and serial analysis of gene expression (SAGE), have resulted in a massive amount of gene sequence and expression information [11]–[19]. However, without reliable reverse or forward genetics methods, this vast amount of data cannot be placed into any functional context that can then be used to determine the value or importance of specific genes as targets for disease control.

Unlike the parasitic nematodes, which have benefited from reverse genetic methods developed in the model free-living worm *Caenorhabditis elegans* [20],[21], no analogous model system is available for schistosomes. This has further delayed the application of new genomic technologies to problems related to disease control and drugs development [22]. However, despite this lack of a *C. elegans*-type model for parasitic flatworms, important advances have been made in trematode transgenesis with the introduction and transient expression of various reporter constructs in schistosomes [22],[23] and fasciolids [24], although these approaches did not permit the functional assessment of specifically introduced genes. With the first demonstrations of gene expression knockdown by RNA interference (RNAi) in the mammalian [25] and snail [26] stages of *S. mansoni*, this reverse-genetics approach has now been applied to a limited number of genes expressed in primary ( = mother) sporocysts [27], schistosomula [28],[29] and adults [30]. In a recent review of RNAi in parasitic helminths, however, Geldhof and collaborators [31] admonish researchers for, at times, providing insufficient data that more firmly connects RNAi-mediated gene expression changes with specific phenotypes, and/or reporting only on genes that are susceptible to double-stranded (ds)RNA-mediated knockdown. Therefore, in order to gain a broader profile of RNAi efficacy in schistosomes, in the current study we performed an *in vitro* phenotypic screening of 32 genes known to be expressed in primary sporocysts of *S. mansoni*. These genes covered a variety of functional categories including antioxidants, transcription factors, cell signaling molecules and metabolic enzymes. Out of the 32 genes (comprising 33 dsRNA treatments) targeted for silencing, one-third (11 genes) exhibited a dsRNA treatment-associated phenotype that consisted of a reduction in sporocyst size (i.e., larval length). Interestingly of the 11 phenotype-yielding genes, only 7 demonstrated a significant and consistent alteration in transcript expression after the 7-day treatment period, although time-course experiments suggest that transient gene knockdown during earlier times of exposure may, in part, account for the observed phenotype.

## Materials and Methods

### *Schistosoma mansoni in vitro* culture and dsRNA treatments

All experiments were performed using the NMRI strain of *S. mansoni*. Eggs were obtained from 7–8 weeks infected mouse livers. After hatching in an artificial “pond water” [32] containing antibiotics (50 µg/mL streptomycin and 60 µg/mL penicillin), miracidia were immobilized on ice for 15 min, washed twice in cold pond water by centrifugation (1 min, 700×g) and gently resuspended in Chernin's balanced saline solution (CBSS) [33], supplemented with glucose and trehalose (1 g/L each), streptomycin (50 µg/mL) and penicillin (60 µg/mL) [34]. Larvae were then counted and distributed into either 48- or 96-well polystyrene tissue culture plates (Costar, Corning Incorporated, NY) at concentrations of ∼6000 and 500 miracidia/well, respectively, depending on the experiments being performed. The general procedure used in all RNAi experiments involve treatment of miracidia starting at day 0 in culture with specific dsRNAs or control media containing irrelevant dsRNAs or medium alone for 7 days followed by assessment of an RNAi-type effect [26]. Details of dsRNA preparation and experimental designs are presented below. All research protocols involving mice used in the course of this study were reviewed and approved by the Institutional Animal Care and Use Committee (IACUC) at the University of Wisconsin-Madison under assurance no. A3368-01.

### Targeted genes

In the present study, a total of 32 genes were selected for quantitative and qualitative assessment. Twenty-three of these genes were chosen based on their abundant expression in *in vitro* cultured *S. mansoni* miracidia and/or primary sporocysts, using the SAGE database OrganismDB [18] http://gmod.mbl.edu/perl/site/s_mansoniest]: calcineurin B (AJ276885.1), lactate dehydrogenase (LDH; U87629.1.), Smad4 (AY371484.1.), Smad2 (AF232025.1), Smad1 (AF215933.1), 14.3.3 (U24281.1), epidermal growth factor receptor (Scmeg; M86399.1), phosphoenolpyruvate carboxykinase (PepCK; AF120929.1), calpain (M74233.1), hexamer-binding protein (HEXBP; putative, organismDB:Tag623), fibrillarin (putative, OrganismDB: Tag 428), elongation factor 1α (EF1α; Y08487.1), Rho 1 GTPase (Rho1; AY158212.1), Rho 2 GTPase (Rho2; AY158214.1), calcium ATPase 2, (Sma2; AF074400.1), SPO1 (AF109181), protein kinase Cß (PKCß; AY337620.1), protein kinase C receptor (PKC receptor; AF422164.1), zinc finger DHHC domain (DHHC; putative, OrganismDB: Tag 1180), myosin-light chain (AF071011.1), calreticulin (L24159.1), high voltage-activated calcium channel subunit α (Cav2A; AF361883.1) and high voltage-activated calcium channel ß-subunit 2 (calcium channel; AY277532.1).

The remaining 9 genes were chosen for their predicted putative functions in the parasite (antioxidants, transcription factors) and ongoing characterization by our group: glycoprotein K5 (AY903301.1), zinc finger 1 (SmZF1; AF316827.1.), ring box (SmRbx; DQ466078.1.), glutathione peroxidase (GPx; M86510.1), thioredoxin peroxidase 1 (TPx-1; AF121199.1), thioredoxin peroxidase 2 (TPx-2; AF157561.1), superoxide dismutase (SOD; M27529.1), 26 kDa glutathione-S-transferase (GST26; M73624.1), and 28 kDa glutathione-S-transferase (GST28; S71584.1). TPx-1 and TPx-2 were used in combination to simultaneously silence both thioredoxin peroxidases. All of the above sequences are available from GenBank (http://www.ncbi.nlm.nih.gov/Genbank) or OrganismDB as indicated above.

### Double-stranded (ds) RNA synthesis

T7 promoter-tagged specific PCR primers were designed to amplify ∼500 base pair (bp) products for each of the targeted genes (Dataset S1; Table 1). A 500-bp green fluorescent protein (GFP) gene segment also was synthesized from the vector pAcGFP (Clontech, Mountain View, CA) to serve as a nonspecific dsRNA treatment-control. Following amplification, PCR products were separated on 1% agarose gels and purified using QIAquick gel extraction kit (Qiagen, Valencia, CA), following the manufacturer's protocol. Each PCR product was sequenced and their sequences verified using the Basic Local Alignment Search Tool (BLASTn, National Center for Biotechnology Information, NCBI). Double-stranded RNAs were synthesized from isolated sporocyst cDNA using T7 RiboMAX Express RNAi Kit (Promega, Madison, WI), according to procedures outlined by the manufacturer. Briefly, dsRNAs synthesis reactions were allowed to incubate for 16 hr at 37°C prior to DNAse treatment. DsRNA products were then phenol/chloroform-extracted and purified by precipitation with isopropanol. The purified products were resuspended in diethylpyrocarbonate (DEPC)-treated water, quantified by measurement at OD_260_ and their integrity verified by 1% agarose gel electrophoresis. Samples were stored at −20°C until further use.

### Phenotypic screening and dsRNA uptake experiments

Effects of dsRNA treatment on *in vitro* cultured *S. mansoni* larvae were performed in 96-well culture plates (Costar) in which approximately 500 miracidia were added to wells containing 50 nM of specific or control green fluorescent protein (GFP) dsRNA diluted in 200 µL CBSS or medium lacking any dsRNA (no dsRNA control). Cultures were maintained at 26°C for 4 days, after which time an additional 10 nM of dsRNA was added to each well due to possible RNA degradation in culture [35], followed by incubation for 3 more days. Over the 7 days culture period, sporocysts were monitored for the following phenotypes: failure/delay in transformation, loss of motility, tegumental lysis and granulation (lethality) and changes in larval growth. Parasite viability and morphological changes were monitored daily using a Nikon Eclipse TE 300 inverted epifluorescent microscope (Nikon Instrument Inc., Melville, NY). In addition digital images of live treated and control parasites were captured using a CoolPix EZ digital camera (Nikon Instruments Inc.) throughout the 7-day incubation period, allowing more detailed observations of larval morphology and to quantify sporocyst growth (length measurements) in treated vs. control larvae at day 7. Length measurements from captured images were obtained and analyzed by Metamorph software version 7.0 (Meta Imaging series, Molecular Devices, Sunnyvale, CA). Sporocysts exhibiting tegumental lysis or loss of surface/somatic integrity were excluded from measurements. Larval growth datasets for each experimental replicate were statistically analyzed using the Mann-Whitney *U*-test (Wilcoxon-Sum of Ranks test) with significance set at P≤0.05. All treatments were performed in duplicate wells, and the experiment was independently replicated a minimum of 3 times on miracidia isolated from different batches of infected mouse livers. In addition, to verify dsRNA uptake by sporocysts we labeled Smad4, lactate dehydrogenase and GFP (specificity control) dsRNAs with rhodamine using the Label IT kit (CX-Rhodamine Labeling Kit; Mirus, Madison, WI), according to manufacturer's recommendations. Miracidia were *in vitro*-transformed to sporocysts in CBSS containing 50 nM labeled dsRNAs and subjected to epifluorescence photomicrography after 7 days of incubation.

### Effect of dsRNA treatment on larval gene expression

In order to demonstrate an association between phenotype and transcript expression real-time quantitative PCR (q-PCR) was used to determine steady-state transcript levels in specific dsRNA-treated sporocysts. In these experiments ∼6000 miracidia were distributed into a 48-well plate (Costar) and treated with 50 nM dsRNA diluted in CBSS (500 µL/well). Cultures were maintained at 26°C for 2, 4 or 7 days prior RNA extraction and isolation. Cultures maintained for 7 days were supplemented with 10 nM dsRNA at day 4. Sporocysts were extensively washed with CBSS in order to eliminate unabsorbed dsRNAs and shed ciliary epidermal plates, followed by extraction in Trizol reagent (Invitrogen, Carlsbad, CA) to isolate both total RNA and protein fractions from cultured larvae. The protein pellet was dissolved in the protein solubilization buffer (3 M Urea, 2% CHAPS, 40 mM Tris) for use in Western blot analyses (see below), while the isolated RNA fraction was resuspended in DEPC-treated water and subjected to DNAse treatment using the DNA-Free kit (Ambion, Austin, TX) to eliminate any contaminating genomic DNA. RNA samples were quantified and their purity assessed on a Nanodrop Spectometer ND-1000 (NanoDrop Technologies, Inc., Wilmington, DE).

### Real-time quantitative PCR (q-PCR) analysis

Quantitative PCR analysis was used to compare steady-state transcript levels between specific dsRNA-treated sporocysts and control treatments (GFP-dsRNA). To accomplish this 0.5 to 1 µg total RNA, derived from at least three different extractions, was used to synthesize cDNA using Superscript III cDNA Synthesis kit (Invitrogen, Carlsbad, CA) following the manufacturer's protocol. The q-PCR reaction mixtures consisting of 2.5 µL of cDNA, 12.5 µL of Sybr Green PCR Master Mix (Applied Biosystems, Foster City, CA), 10 µL of 600 or 900 nM primers (determined after primer concentration optimization), were added to 96-Well Optical Reaction Plates (ABI PRISM, Applied Biosystems) for amplification and quantification in a AB7500 Real Time PCR System (Applied Biosystems). In order to avoid the possibility of false amplification of the originally applied dsRNA, specific pairs of primers were designed outside of the region used to synthesize the original interfering dsRNA products (Dataset S1; Table 2). In addition to the targeted gene-specific primers used to assess potential knockdown, primers for *S. mansoni* glyceraldehyde 3-phosphate dehydrogenase (GAPDH) and α-tubulin were used as endogenous normalization controls in all samples tested. Other controls for verifying the specificity of RNA treatment effects included (1) larval treatment with irrelevant GFP dsRNA and (2) treatment with a nontarget *S. mansoni* dsRNA. Finally, each q-PCR run was performed with 2 internal controls assessing both potential genomic DNA contaminations (no reverse transcriptase added) and purity of the reagents used (no cDNA added). For each specific set of primers, all individual treatments (including specificity controls) were run in three technical replicates. Each experiment was repeated 3–5 times (N = 3–5) as independent biological replicates and the ΔΔCt method [36], using GAPDH and α-tubulin as endogenous loading controls to normalize the quantification of all cDNA targets was used to quantitatively compare treatment and control steady-state transcript levels. Due to the nonparametric distribution of data, statistical analysis of ΔΔCt values was performed using the Mann-Whitney *U*-test with significance set at *P*≤0.05.

### Effect of specific dsRNA treatment at the protein level

Using a sporocyst-reactive rabbit anti-elongation factor 1α (anti-EF1α) antibody (Cell Signaling Technology, Danvers, MA), Western blot analysis and immunocytochemical localization experiments were performed to monitor EF1α protein levels in EF1α dsRNA-treated sporocysts. For Western blots miracidia, transformed in the presence of 50 nM EF1α or control GFP dsRNA and cultivated *in vitro* for 7 days, were extracted in Trizol reagent (Invitrogen) as previously described, separated by standard 12.5% SDS-PAGE methods [37], and electroblotted to nitrocellulose membranes (Biorad Lab, Richmond, CA) using a semi-dry protein transfer apparatus (Hoefer TE 70, Amersham Biosciences). After transfer, membranes were blocked overnight in TBS (2.42 g Tris base and 8 g NaCl/L, pH 7.6) containing 5% bovine serum albumin (BSA), followed by incubation in a mixture of anti-EF1α (1∶ 1000) and rabbit anti-SmGST26 (loading control, 1∶1000 dilution; Cell Signaling Technology), for 16 hr at 4°C. Membranes were washed 3 times in TBS-Tween (0.1%) and incubated for 1 hr in TBS 5% BSA containing either goat anti-rabbit IgG-alkaline phosphatase-tagged (AP) or AP-labeled goat anti-rabbit IgG (1∶10^4^ and 1∶5000, respectively). Colorimetric immunoreactivity was detected with the chromogen substrate 5-bromo-4-chloro-3-indolyl phosphate (BCIP) and nitro-blue tetrazolium (NBT), diluted in AP buffer (0.1 M Tris, 0.1 M NaCl, 0.05 M MgCl_2_, pH 9.5). To quantify the relative levels of anti-EF1α in specific dsRNA- and control GFP dsRNA-treated sporocyst extracts, target and control immunoreactivities were measured using an Ultraviolet Trans-illuminator BioImaging Systems (UVP, Inc., Upland, CA) with the co-processed anti-GST26 band serving as a normalizing signal (loading control). Quantitative comparisons of protein expression for EF1α in control and target dsRNA-treated sporocysts were analyzed by LabWorks Image Acquisition and Analysis Software version 4.6.

For immunocytochemical studies, 7-day old dsRNA-EF1α or -GFP-treated sporocysts were washed in CBSS (allowing removal of ciliated epidermal plates), transferred to siliconized-microcentrifuge tubes in 500 µL PT buffer (2% paraformaldehyde, 1% Triton-X100/sPBS), and incubated overnight at 4°C with constant rotation. Fixed-parasites were washed 5 times in sPBS by centrifugation at 1600 rpm (2 min), resuspended in 500 µL of blocking buffer (5% normal goat serum/0.02% azide/sPBS) for 16 hr, under constant agitation before addition of rabbit-anti-EF1α primary antibodies (1∶200 dilution in blocking buffer) and incubation overnight at 4°C. Parasites were washed for 10 min in sPBS, resuspended in 500 µL of AlexaFluor 488-conjugated goat anti-rabbit IgG (4 µg/mL blocking buffer) and incubated for 16 hr at 4°C with agitation. Following antibody treatments, sporocysts were washed 5 times in sPBS by centrifugation (1600 rpm, 2 min), resuspended in 40 µL of sPBS and mounted on coverslips. Specimens were examined and photographed using a Nikon Eclipse TE2000 (Nikon Instrument Inc.) inverted microscope equipped with a Bio-Rad Radiance 2100 MP Rainbow Confocal/Multiphoton Imaging System (W.M. Keck for Biological Imaging, Instrumentation, UW-Medical School).

## Results

**Figure 1:**
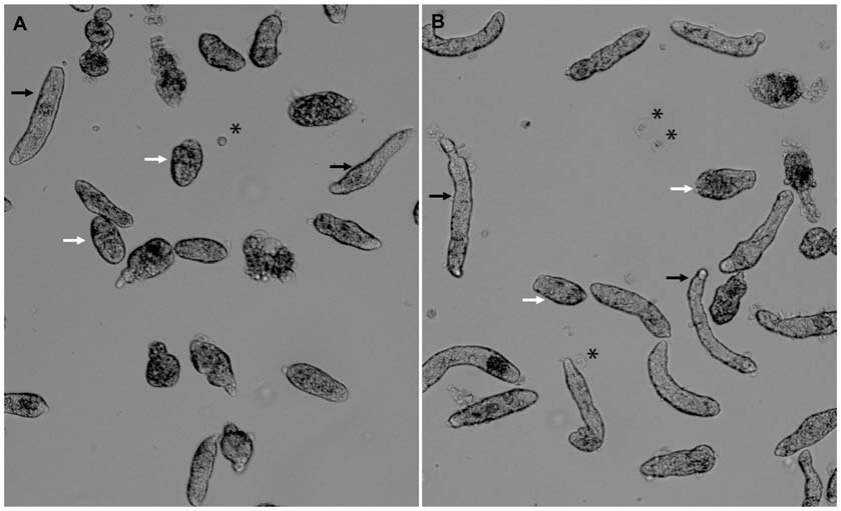
*In vitro* cultured *S. mansoni* larvae 7 days post-dsRNA treatments. Brightfield photomicrographs of *in vitro* cultured *Schistosoma mansoni* sporocysts after 7 days of treatments with a specific GST26-dsRNA (A) compared to the control GFP-dsRNA (B), illustrating the effects of exposure to phenotype-inducing GST26-dsRNA on sporocyst lengths. Arrows indicate examples of shortened (white arrows) and normal elongate (black arrows) sporocysts measured in both treatments. Asterisks indicate rounded ciliated epidermal plates that were shed from the miracidial surface after transformation.

**Figure 2:**
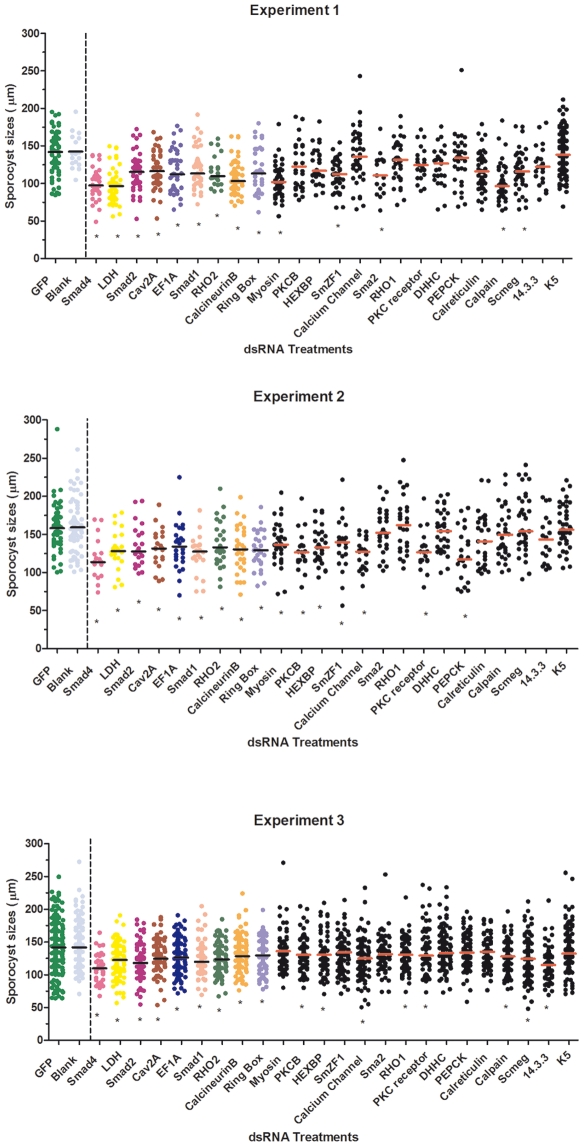
*S. mansoni* sporocyst length measurements post-dsRNA treatments. Graphic representation of sporocyst length measurements (µm) after 7 days of dsRNA treatments, from 3 independent experiments (A–C). Sporocyst length measurements are represented by scatter plots with the calculated median values indicated by the horizontal bars within each dsRNA treatment. The median values for specific dsRNA treatments were compared to both GFP-dsRNA (green plots) and blank (no dsRNA; blue plots) treatment controls. For each of the 3 experiments, the pair of controls is shown in between solid and dashed vertical black lines, immediately followed by the gene-specific dsRNAs groups. The dsRNA treatments exhibiting significant differences from GFP and blank controls in all 3 replicate experiments are represented as colored plots, marked with an asterisk (Smad4, lactate dehydrogenase, Smad2, Cav2A, EF1α, Smad1, RHO2, calcineurin B, and ring box), while those yielding inconsistent phenotypic differences when compared to the controls (myosin, PKCB, HEXBP, SmZF1, calcium channel, Sma2, RHO1, PKC receptor, DHHC, Pepck, calreticulin, calpain, Smeg, 14.3.3, and K5) are indicated by black dot scatter plots. Of this latter group, asterisks denote those individual replicates that were significantly different from controls. All treatments were statistical analyzed using Mann-Whitney *U*-test within each experiment, *P≤0.05.

**Figure 3:**
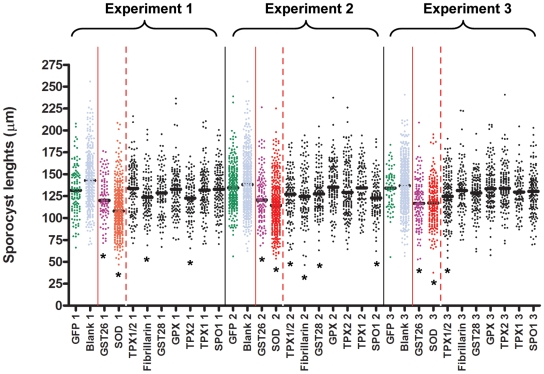
Additional *S. mansoni* sporocyst length measurements 7 days post-dsRNA treatments. Graphic representation of sporocyst lengths (µm) after 7 days post-dsRNA treatments, generated from 3 independent experiments covering an additional group of sporocyst-expressed genes. Larva lengths are represented by a dot scatter plots with the median length for each sporocyst treatment group shown as a short horizontal bar. Calculated medians for each target dsRNA group were compared to both GFP-dsRNA (green dots) and no-dsRNA (blank, blue dots) control median values. For each experiment, controls are the first 2 scatter plots shown between the solid red and black vertical lines, followed by the 2 dsRNA treatments that showed significant phenotypic differences in all 3 experiments (marked with *; GST26 and SOD) when compared to both controls. Black scatter plots represent dsRNA-treated sporocysts whose median length measurements exhibited inconsistent differences when compared to both GFP and blank controls. Of this latter group (TPx1/2, fibrillarin, GST28, GPx, TPx2, TPx1, and SPO1), asterisks denote those individual replicates that were significantly different from controls. Each experiment was analyzed using Mann-Whitney *U*-test, *P≤0.05.

In our initial phenotype analysis of 24 different dsRNA treatments, no differences between specific dsRNA-treated larvae and controls (GFP dsRNA-treated and untreated sporocysts) were noted in miracidial transformation rates, larval motility or mortality during the *in vitro* cultivation period. The only notable phenotype observed in treated 7-day cultured sporocysts was an apparent greater number of small-sized or shortened sporocysts possibly involving a growth-related defect(s) (Fig. 1A, 1B). However, because sporocysts in a given culture population typically represented a range of sizes, live sporocyst images were captured, from which larval lengths were measured and digitally-analyzed using Metamorph software. Within each biological replicate, such measurements were taken for the dsRNA treatment groups and statistically compared to both the GFP dsRNA-treated and no treatment (blank) groups. For a given dsRNA to be identified as having a putative dsRNA-mediated effect, the median larval length had to significantly differ (*P*≤0.05) from both the GFP dsRNA and the blank controls in each of the biological replicates. Using these criteria, we observed significant decreases in parasite lengths in 9 of 24 dsRNAs in the first screening trial: Smad4, lactate dehydrogenase (LDH), Smad2, Cav2A, elongation factor 1α (EF1α), Smad1, RHO2, calcineurin B, and ring box (Fig. 2). Similar results were found in a second experimental series, which included nine additional dsRNA treatments. In this case, using the same criteria for significance, 2 of the 9 dsRNAs treatments (GST26 and SOD) exhibited a consistent size-related phenotype effect when compared to controls (Fig. 3). As before, greater frequency of shortened larvae was the only observable dsRNA-associated phenotype.

To illustrate the variability in parasite response to the different dsRNA at the population level, and to underscore the importance of biological replication in phenotypic analysis of dsRNA treatment effects, 9 of the 33 dsRNA treatments (Scmeg, HEXPB, Sm zinc finger1 (SmZF1), -calpain, -myosin light chain, PKCß, SPO1, TPx1/2 and calcium channel) exhibited significant length decreases in 2 of 3 experiments suggesting a possible, but inconsistent, connection with the observed phenotype. Sporocyst treatment by the remaining dsRNAs (14-3-3 protein, Sma2, RHO1, PKC receptor, DHHC, PEPCK, calreticulin, glycoprotein K5, fibrillarin, GST28, GPx, TPx1, and TPx2) had no measurable effect on larval phenotype (Figs. 2 and 3).

**Figure 4:**
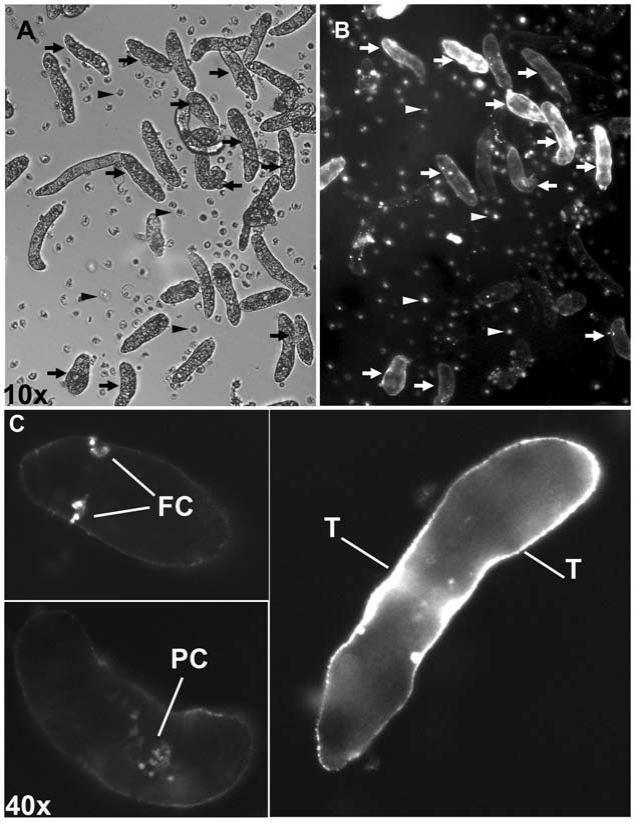
Localization of rhodamine-labeled-dsRNA in *S. mansoni* larvae 7 days post-exposure. Brightfield (A) and fluorescent (B) photomicrographs showing *S. mansoni* sporocysts and localization of rhodamine-dsRNA taken up after 7 days of labeled dsRNA exposure, respectively (100×). Arrowheads indicate rounded epidermal plates that were shed from miracidia during transformation to sporocysts. (B) Fluorescent images show the different levels of dsRNA penetrance within the same treatment and the same population. (C) The higher magnification (400×) illustrating the heterogeneity of dsRNA uptake within individual sporocysts in a given population including excretory ducts/flame cells (FC), cells within the parenchyma (PC), and tegument (T).

In order to document potential difference in dsRNA uptake within larval populations and between treatments, miracidia were exposed to rhodamine (Rh)-labeled dsRNA-GFP, -Smad4 or –LDH. After 7 days of exposure, dsRNA-uptake in sporocysts was assessed by fluorescent microscopy. As shown in Figure 4, larvae within a single population exhibited wide variation in their abilities to take up labeled dsRNA, regardless of transcript species (Figs. 4A and B). Indeed, a one-way ANOVA comparing the 3 dsRNA-treated groups for the prevalence of tegumental or internal staining was non-significant (F = 1.159; *P* = 0.2555) indicating no differences in staining distribution between groups or locations. The most prominent sites of Rh-dsRNA localization in positively-stained sporocysts (67% of larvae) were in the tegument (∼28%), excretory pores/flame cells and in unidentified parenchymal-like cells (∼39%) (Fig. 4C). Negative controls consisting of larvae treated with unlabeled-dsRNA did not display any fluorescent signal (data not shown). Sporocysts within a given population exhibited heterogeneous (+/−) Rh-staining indicating specific dsRNA uptake by larvae, and not a nonspecific uptake via Rh-binding.

**Figure 5:**
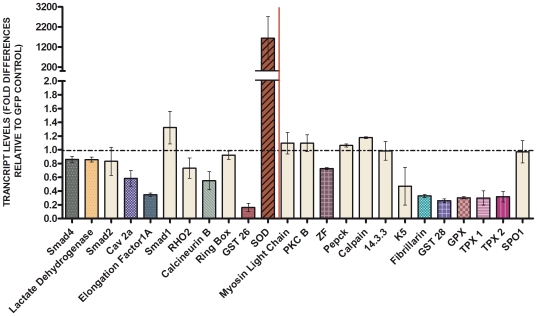
Transcript levels of dsRNA-treated sporocysts 7 days after dsRNA exposure. Bar graph depicting the relative steady-state transcript levels of dsRNAs-treated sporocysts after 7 days of exposure compared to the GFP-dsRNA control. For each dsRNA tested, data are represented as mean fold-differences (+/−S.E.) relative to the GFP control (1.00). Colored bars represent sporocyst mRNA levels showing consistent and statistically significant decrease (dsRNA-Smad4/GFP, *P* = 0.0056; -lactate dehydrogenase/GFP, *P* = 0.0358; -Cav2A/GFP, *P* = 0.0136; -EF1α/GFP, *P* = 0.0358; -calcineurin B/GFP, *P* = 0.0189; -GST26/GFP, *P* = 0.0136; -SmZF1/GFP, *P* = 0.0189; -fibrillarin/GFP, *P* = 0.0407; -GST28/GFP, *P* = 0.0284; -GPx/GFP, *P* = 0.0269 and -TPx1/GFP, *P* = 0.0358/-TPx2/GFP, *P* = 0.0358) or increase (dsRNA-SOD/GFP, *P* = 0.0294) in target transcript levels when compared to the GFP-dsRNA control treatment. Tan-colored bars represent transcript levels for dsRNA-treated sporocysts that showed no differences when compared to GFP-dsRNA treated controls (-Smad2/GFP, *P* = 0.0755; -Smad1/GFP, *P* = 0.8969; -RHO2/GFP, *P* = 0.0765; -ring box/GFP, *P* = 0.7642; -myosin/GFP, *P* = 0.3725; -PKCB/GFP, *P* = 0.6579; -PEPCK/GFP, *P* = 0.3017; -calpain/GFP, *P* = 0.1642; -14.3.3/GFP, *P* = 0.6579; -K5/GFP, *P* = 0.3725 and -SPO1/GFP, *P* = 0.8969). In addition, bars located on the left of the solid red vertical line represent treated-sporocysts previously shown to express the shortened phenotype (dsRNA-Smad4, -lactate dehydrogenase, -Smad2, -Cav2A, -EF1α, -Smad1, -RHO2, -calcineurin B, -ring box, -GST26 and -SOD). Transcript levels were determined by q-PCR and data analyzed using the ΔΔCt method [36] followed by statistical analysis using the Mann-Whitney *U*-test. Significance levels were set at *P*≤0.05. Data were generated from 3–5 independent experiments.

Because the phenotypic screen revealed both phenotype-associated and nonphenotype-associated dsRNA species, we selected a subset of 24 genes to assess the effect of dsRNA treatments on steady-state transcript levels using real-time quantitative PCR (q-PCR). Comparisons of normalized-levels of dsRNA-targeted messenger RNAs to their corresponding control treatment (GFP dsRNA-treated group) resulted in 12 transcripts that exhibited significantly reduced expression levels (Fig. 5). Unexpectedly, SOD transcripts consistently increased, rather than decreased, to very high levels of expression (>1600-fold) upon specific dsRNA treatment.

A comparison was made between dsRNA species that produced a detectable phenotype and those generating a significant transcript knockdown (or induced expression) in an attempt to directly correlate phenotype and gene expression. Notably, only 7 of the 11 target dsRNA-treatments that produced a “shortened” larval phenotype presented a significant alteration in transcript levels (Smad4, lactate dehydrogenase, Cav2A, EF1α, calcineurin B, GST26 and SOD) when compared to dsRNA-GFP treated sporocysts (Fig. 5). Smad4 and LDH dsRNA treatments showed a small, but consistently significant 15% decrease, while Cav2A, calcineurin B, EF1α and GST26 exhibited knockdowns of 42%, 65%, 70% and 85%, respectively. SOD, whose transcript levels were dramatically increased in dsRNA-treated sporocysts, also was phenotype-associated. In addition, as noted in Figure 5, 6 dsRNA treatment groups that did not exhibit significant or consistent changes in larval length expressed significantly lower transcript levels than controls ranging from an approximately 30% (SmZF1) to 75% (fibrillarin, GST28, GPx, TPx1, and TPx2) after 7 days of exposure. No changes in transcript levels were observed for phenotype-associated Smad1, Smad2, RHO2 and ring box dsRNA treatments and phenotype-non-associated myosin, PKCB, Pepck, calpain, 14.3.3 protein, glycoprotein K5, and SPO1 dsRNAs.

**Figure 6:**
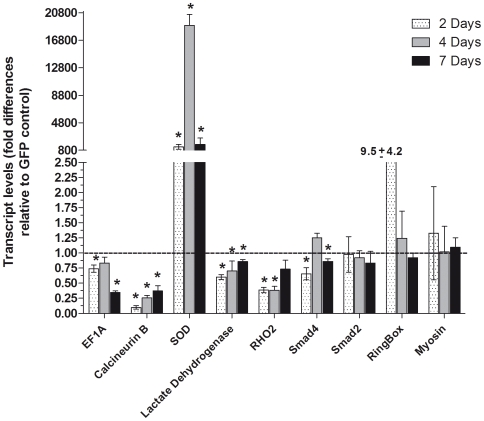
Transcript levels in *S. mansoni* sporocysts at different times post-dsRNA treatment. Time-course of steady-state transcript levels was assessed in sporocysts treated with dsRNAs under culture conditions. Sporocysts were treated with dsRNA-EF1α, -calcineurin B, -SOD, -lactate dehydrogenase, -RHO2, Smad2, -Smad4, -myosin and -ring box for 2 days (stippled bars) or 4 days (gray bars), and compared to 7 day dsRNA treatment effects (black bars). Transcript levels were assessed by q-PCR at each time and compared to its matched GFP-dsRNA control. For each dsRNA tested, data are represented as mean fold-difference (+/−S.E.) relative to the GFP control (1.00). However, statistical analyses were based on raw q-PCR values using the ΔΔCt method followed by statistical analysis using the Mann-Whitney *U*-test, N = 4, *P≤0.05. Two-day comparisons (stippled bars): dsRNA-EF1a/GFP, *P* = 0.028; -calcineurin B/GFP, *P* = 0.021; -SOD/GFP, *P* = 0.015; -lactate dehydrogenase/GFP, *P* = 0.041; - RHO2/GFP, *P* = 0.021; –Smad4/GFP, *P* = 0.028; -ring box/GFP, *P* = 0.3; -Smad2/GFP, *P* = 1.0; and -myosin/GFP, *P* = 0.059. Four-day comparisons (gray bars): dsRNA-EF1a/GFP, *P* = 0.0319; -calcineurin B/GFP, *P* = 0.03; -SOD/GFP, *P* = 0.028; -lactate dehydrogenase/GFP, *P* = 0.029; - RHO2/GFP, *P* = 0.021; –Smad4/GFP, *P* = 0.0286; -ring box/GFP, *P* = 0.884; -Smad2/GFP, *P* = 0.98; and -myosin/GFP, *P* = 0.9. Data for 7 day dsRNA treatments (black bars) were taken from identically performed experiments (data shown previously in Fig. 5), and are reproduced in Fig. 6 for graphic comparisons only. Statistics for this group of genes are provided in the Fig. 5 legend.

Since we typically used day 7 as our temporal end-point for assessing RNAi phenotypic effect, we also investigated the possibility that transcripts may have been knocked down prior to day 7. Using a subsampling of dsRNA species that represented a range of transcript knockdown levels, *S. mansoni* miracidia were treated with dsRNA-EF1α, -calcineurin B, -SOD, -LDH, -RHO2, -Smad2 -Smad4, myosin light chain and -ring box, and sporocyst transcript levels analyzed after 2 and 4 days postexposure. Compared to our previous 7-day treatment effects, results yielded various patterns of transcript silencing (Fig. 6). For example, although EF1α and calcineurin B transcripts were significantly reduced by ∼70% by day 7, calcineurin B knockdown was actually greatest (∼90%) at 2 days postexposure to dsRNA. Smad4 and LDH mRNAs, which previously showed a small, but significant, decrease at day 7 exhibited highest knockdown (∼40%) on day 2 indicating an early effect of dsRNA treatment. SOD was found to be over-expressed at all time points, with an initial increase of ∼1200% at day 2, followed by a maximum ∼17,000-fold expression at day 4, before again returning to day-2 levels after 7 days of exposure. SOD transcript levels, however, were unaffected by heterologous exposure of larvae to several non-SOD-related dsRNAs (data not shown). RHO2 dsRNA, previously displaying no effect on homologous transcript expression in sporocysts at 7 days, showed significant transcript knockdown at 2 and 4 days post-exposure before recovering to control levels at the 7-day time point. In contrast, Smad2, myosin light chain and ring box dsRNA treatments demonstrated no consistent effect on their respective transcript levels regardless of the sampling interval.

**Figure 7:**
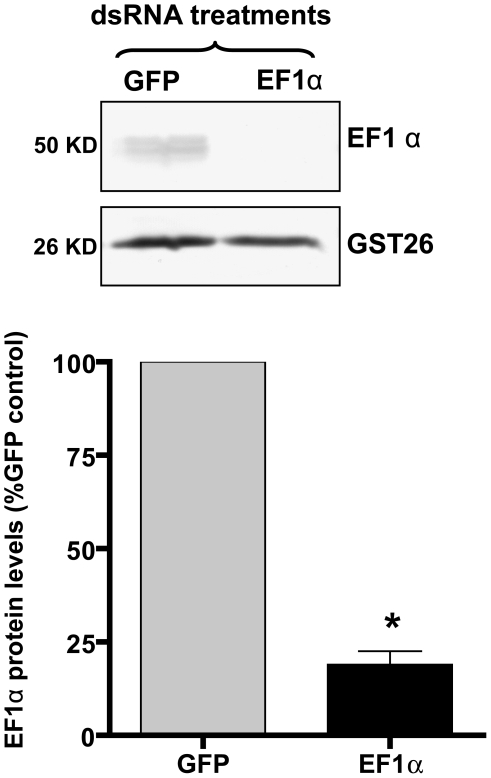
Quantification of differential EF1α protein levels in *S. mansoni* sporocysts post-dsRNA exposure. Western blots of SDS-PAGE separated total proteins extracted from sporocysts treated for 7 days with elongation factor1α (EF1α) or GFP (control) dsRNA. A rabbit anti-EF1α was used to detect a 50 kDa *S. mansoni* EF1α, while a rabbit anti-SmGST26 antibody served as both a protein specificity and loading control. Note the presence of EF1α protein in dsRNA-GFP treated-sporocysts, but reduced reactivity in EF1α-dsRNA-silenced parasites. Significant knockdown of EF1α protein in EF1α dsRNA-treated parasites was confirmed by optical densitometry comparing protein band intensities of test and control dsRNA treatment groups after anti-GST26 normalization of each band. The bar graph shows an 80% reduction (+/−S.E.) in dsRNA-induced EF1α protein level in EF1α dsRNA-treated sporocysts relative to GFP controls. Statistical analyses were performed using Students *t*-test. *P≤0.05; N = 3.

**Figure 8:**
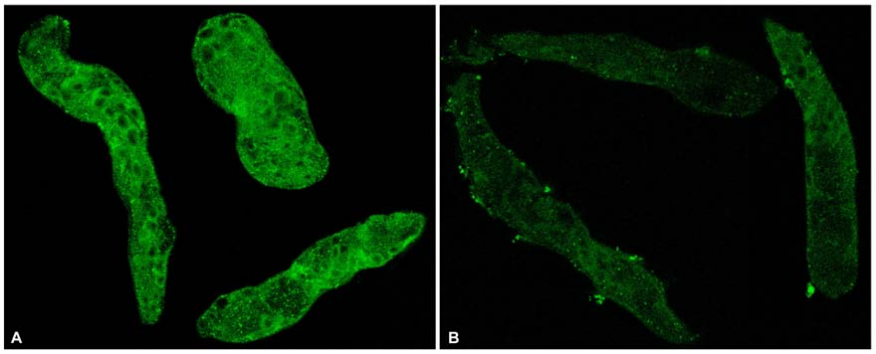
Observations of differential expression of EF1α protein levels in *S. mansoni* sporocysts after dsRNA treatments. Immunfluorescence photomicrographs of GFP dsRNA (control; A) and EF1α dsRNA (B) treated sporocysts showing EF1α protein-knockdown post RNAi treatments. Larvae were cultured with dsRNAs for 7 days and fixed prior to treatment with anti-EF1α antibody and Alexa 488-conjugated secondary antibody. Strong immunoreactivity (green fluorescence) is distributed among various cells and tissues within interior of control sporocysts (A), compared to only weak reactivity in EF1α dsRNA-treated sporocysts (B). Confocal images; 400×. N = 2.

Finally, because we had available an antibody that was specifically reactive to the *S. mansoni* EF1α protein, we assessed the effect of EF1α dsRNA treatment on EF1α protein levels using Western blot and immunofluorescence imaging. Western blot analysis clearly showed that EF1α dsRNA-treated 7-day sporocyst extracts were significantly reduced in EF1α protein (50 kDa band) compared to the GFP dsRNA-treated control group (Fig. 7). The presence of a 25 kDa GST26 band (used as an antibody specificity and loading control) in both the EF1α and GFP dsRNA-treated samples suggested both a specific EF1α transcript silencing and associated protein knockout (Fig. 7). This result was quantitatively confirmed by densitometry showing that, following normalization of transcripts to the loading control, EF1α protein was highly reduced by >80% in the EF1α dsRNA-treated sample compared to the GFP dsRNA control. Confocal immunolocalization of EF1α in intact dsRNA-treated sporocysts was consistent with the Western blot analysis: EF1α dsRNA-treated larvae displayed little immunoreactivity, while abundant anti-EF1α-reactivity was evident within cells and parenchymal tissues of GFP dsRNA-treated sporocysts (Fig. 8).

## Discussion

RNA interference (RNAi) has been widely used in a variety of organisms as a reverse-genetic approach to generate functional gene knockdowns with associated phenotypic changes [38]–[40]. In combination with complete and well-annotated genome databases, tools developed for RNAi now permit systematic, whole-genome screening leading to putative functional assignments for unknown genes, or direct functional confirmation of genes identified by sequence homology (orthologues) [41]. Some RNAi libraries are already available and have taken advantage of this functional genomics approach including for *D. melanogaster* [39] and *C. elegans* [42],[43]. However, many newly-defined functions for any given gene tend to be organism-specific and may not always be identical to, or even homologous with, a similar gene's function in other species. Therefore, one of the current challenges we face is to integrate this organism-specific RNAi-derived functional information into the existing, ever-growing genomic databases of diverse organisms [44]. In addition, as noted by Geldhof [31], application of RNAi approaches to parasitic helminths have at times lacked convincing evidence of an RNAi effect or have not provided information on the full spectrum or diversity of target transcript susceptibilities to dsRNA treatments.

The RNAi screening approach described in the current study, to our knowledge, is the first to profile morphological phenotypes associated with exposure of larval schistosome blood flukes to dsRNAs representing a diversity of expressed genes. From our sampling of dsRNAs for 32 different *S. mansoni* genes known to be expressed in primary sporocysts, only 34% (11 transcripts) produced a consistent, highly reproducible phenotype; namely a reduced larval length (shortening), morphologically resembling a type of growth inhibition. Interestingly, this was the same phenotype that was observed in an earlier study involving dsRNA-mediated knockdown of a CD36-like scavenger receptor at the tegumental surface of *S. mansoni* sporocysts [27]. Thus, the genes associated with this phenotype are quite varied, including signaling molecules, Ca-interactive proteins, redox enzymes and a membrane receptors/ion channels. Although it would be premature to speculate on specific gene-phenotype linkages, it may not be particularly surprising that such a general phenotype as larval size might be regulated by many different genes expressed in variety of cell or tissue-types. In whole genome RNAi studies of *C. elegans*, an overall ∼2% of detectable viable phenotypes were growth- or size-related [41] and recent RNAi applications on schistosomules, miracidia, or adults also produced a similar consistent “shorter” phenotypes [45]. Our finding that only a proportion of sporocyst exhibited the shortened phenotype might be explained, at least in part, by results of the rhodamine-labeled dsRNA uptake experiment demonstrating that ∼67% of dsRNA-treated larvae within a population (in all treatments) exhibited signs of labeling, and of those, cellular localization of Rh-dsRNA within sporocysts varied considerably (tegument, flame cells, parenchymal tissues). Thus the degree and site of dsRNA penetrance may be among several critical determinants influencing the observed phenotype. Moreover, this differential dsRNA uptake also could explain the variation in the levels of transcript knockdown observed in q-PCR analyses.

Attempts to correlate phenotype and knockdown of target gene expression also yielded variable results in that 7 of the 11 genes associated with the shortened phenotype were significantly altered in their expression after the treatment period. One explanation as to why all phenotype-expressing transcripts were not reduced is the possibility that some genes possess different kinetic profiles (i.e., may have exhibited knockdown prior to day 7). Of 5 genes whose transcript levels were unaffected (Smad2, Rho2, ring box) or marginally affected (Smad4, LDH) by dsRNA at 7-days of treatment, 3 transcripts (Rho2, Smad4 and LDH) showed an early significant knockdown at day 2 suggesting a temporal reduction in transcript levels that could be phenotype-associated. Why transcript expression of the other 2 phenotype-associated genes (Smad2 and ring box) was not affected by specific dsRNA treatments remains unclear, although RNAi off-targeting, i.e., a mis-targeting of specific dsRNA to other unidentified mRNAs [46]–[49] could be involved. Off-target effect of introduced dsRNAs seems to be a common occurrence in helminth RNAi experiments, and presents a challenge in controlling such effects, as recently reviewed by Geldhof and colleagues [31]. Yet, it has been shown in mammal cells that knockdown efficiency is highly dependent on the specific dsRNA sequence of a particular gene [50], and that in some cases, a small degree of similarity may invoke off-target gene silencing [51]. In our current study, we exposed larvae to gene-specific long (500 bp) dsRNA, which, upon dicer cleavage, results in short unpredictable RNA sequences that represent potential sources of off-target gene silencing. To complicate matters further, some siRNA also have been shown to exhibit nonspecific toxic effects that may directly affect transcriptional processes without altering specific transcript levels [52].

Although it was not the goal of this study to provide in-depth analyses of each the gene investigated herein, followup experiments involving *S. mansoni* elongation factor 1α (EF1α) illustrates the importance of providing several lines of evidence of an RNAi effect. In this case, larval treatment with EF1α dsRNA resulted in a demonstrable phenotype, specific transcript knockdown, and approximately 80% inhibition of EF1α protein expression as measured by both Western blot and immunocytochemical assays. Elongation factors are known to be essential in the translational process by functioning to catalyze the aminoacyl-tRNA delivery to ribosomes during protein elongation [53]. Given this putative function of EF1α, and its widespread knockdown at the transcript and protein levels, its involvement either directly or indirectly in generating the shortened larval phenotype is supported by the data presented here. Reasons why we did not see any changes in GST26 protein levels in the EF1α Western blot assay maybe due to a slow protein turnover rate for GST26 or the possibility that GST26 is synthesized in cells/tissues that were unaffected by EF1α dsRNA knockdown.

Variation in dsRNA treatment effects also was evident in the group of sporocysts that lacked any definable phenotype. Of the 13 nonphenotype-expressing larval groups, half (6) exhibited consistent, significant reductions in transcript levels as measured by q-PCR, while transcript levels in the other half (7) were unaffected. This type of result is not unexpected as this has been demonstrated previously in RNAi screens of model organisms such as *C. elegans* [54], as well as parasitic nematodes [31],[55]. For those transcripts whose expression was unaffected by specific dsRNA treatment, there would not be an *a priori* expectation of phenotypic change. There are several ways to potentially explain a lack of differential phenotype in larvae presenting with dsRNA-induced transcript knockdown : (1) the gene targeted for dsRNA knockdown is functionally unrelated to the observed phenotype, (2) since typically an RNAi-like effect does not lead to a complete gene (and presumably protein) knockout, sufficient protein synthesis/activity remains to continue support of the normal “phenotype”, (3) other related proteins and/or isoforms may be supplementing or replacing the protein (and its function) initially targeted for dsRNA-mediated silencing, and (4) the protein product of the targeted transcript may have a lengthy half-life (i.e., slow turnover rate), hence delaying potential gene knockdown effects at the protein level. These results further underscore the wide variation in susceptibilities of individual *S. mansoni* genes to RNAi procedures, and the fact that dsRNA knockdown may not be associated with any demonstrable phenotype.

One of the more intriguing results of our study was the consistent, high level upregulated expression of SOD in sporocysts upon treatment with SOD dsRNA. Even more impressive than the 1600-fold transcript expression following the standard 7-day incubation period was the ∼17,000-fold expression 2 days earlier (day 4). At present we do not have an explanation as to how larval exposure to SOD dsRNA may be triggering such high expression levels. One possibility is that the yet unknown sequence(s) in processed SOD dsRNA may be stimulating reactions similar to RNA activation (RNAa) [56],[57]. If this is indeed the case, this would be a novel facet of RNAi in parasitic helminths. Although the function of SOD as a protective anti-oxidant has been suggested [58]–[60], its essential role in parasite development has not been established. If the overexpression response seen in this study is linked to SOD depletion or SOD sequence activation, this would imply a critical role in sporocyst survival, and perhaps in larval development as evidenced by its association with the sporocyst size phenotype. The role of endogenous SOD, and other anti-oxidants, in sporocysts confronted with oxidative stress is the subject of ongoing investigations in our laboratory.

To date, RNAi is the only reverse genetic tool available in schistosomes [22], and although it has been successfully applied as a functional genomics tool in both mammalian [25],[28],[61] and snail [26],[27] stages of infection, a lack of consistency in the RNAi-induced knockdown and resulting phenotypes indicates a pressing need to more fully investigate RNAi to gain a better understanding of this complex mechanism in *S. mansoni*, and other parasitic flatworm species [31]. The results presented here provide an overview of the variability that may be encountered as transcript-specific dsRNA sequences are applied as a tool for targeted gene manipulation and morphological phenotyping in larval schistosomes. It is anticipated that further improvements in dsRNA delivery methods likely would be beneficial in attaining more consistent transcript/protein knockdowns and resulting phenotypes, as will further detailed analyses of specific siRNA for individual genes. Future studies involving optimization of transfection reagent- and electroporation-based gene delivery approaches are currently being planned. In assessing RNAi effects, in addition to low and/or inconsistent dsRNA penetrance, we also are hindered by the numbers of parasites that can be processed for dsRNA treatments at a given time, and, as reported in this study, a very limited phenotype repertoire, due to a lack of more sensitive detection tools. These restrictions illustrate some of the limitations facing large-scale RNAi experiments, and demonstrate the necessity of small-scale or gene-by-gene characterizations, until development of more sensitive, higher-throughput methodologies [44].

In summary, this study is the first to provide a multi-gene assessment of the efficacy of dsRNA treatments in characterizing phenotypic and transcriptional changes brought about by introduction of gene-specific dsRNAs into cultured *S. mansoni* larvae. Prolonged exposure to dsRNA, when selectively applied to target genes expressed in early larval stages, can generate significant transcript knockdown, thus facilitating the investigation of potential gene-associated function. However, as shown in the present study, individual genes may differ significantly in their abilities to render RNAi-like effects, and this is likely due, at least partially, to efficacy of their intracellular processing. Although RNAi approaches continue to be potentially valuable tools for functional genomics in parasitic helminths, caution should be taken in the design, set-up and execution of RNAi experiments. As a followup to this study, we are now focusing on the group of enzymes involved in reduction-oxidation (redox) reactions, especially those with antioxidant activity, and that have exhibited consistent transcriptional knockdown by RNAi. These functional studies were made possible by the data provided in this initial multi-gene profiling of dsRNA effects.

## Supporting Information

Dataset S1 Target genes and primers. Table 1: List of genes targeted in the RNAi screening, includes specific forward and reverse primers used to amplify the 500 bp templates for dsRNA synthesis. Also, protein functions were included in the context of *S. mansoni* when possible. Gb: GenBank. Table 2: List of primers used to quantify specific transcripts during real-time Q-PCR analysis.
(0.12 MB DOC)
