# Correction: Phenotypic Screen of Early-Developing Larvae of the Blood Fluke, *Schistosoma mansoni*, using RNA Interference

**DOI:** 10.1371/annotation/ffd29b50-4102-46b9-b6a1-cdb4984df278

**Published:** 2009-09-23

**Authors:** Marina de Moraes Mourão, Nathalie Dinguirard, Glória R. Franco, Timothy P. Yoshino

The name of first author Marina de Moraes Mourão was written incorrectly in the citation. The correct name is: Mourão MM. The correct citation is: Mourão MM, Dinguirard N, Franco GR, Yoshino TP (2009) Phenotypic Screen of Early-Developing Larvae of the Blood Fluke, Schistosoma mansoni, using RNA Interference. PLoS Negl Trop Dis 3(8): e502. doi:10.1371/journal.pntd.0000502.

