# Supplementary material for: Phenotypic Screen of Early-Developing Larvae of the Blood Fluke, Schistosoma mansoni, using RNA Interference
Source: PLoS Negl Trop Dis. 2009 Aug 11;3(8):e502. doi: 10.1371/journal.pntd.0000502 (PMC2719580; doi:10.1371/journal.pntd.0000502)
Supplement: Dataset S1 — Target genes and primers. Table 1: List of genes targeted in the RNAi screening, includes specific forward and reverse primers used to amplify the 500 bp templates for dsRNA synthesis. Also, protein functions were included in the context of S. mansoni when possible. Gb: GenBank. Table 2: List of primers used to quantify specific transcripts during real-time Q-PCR analysis. (0.12 MB DOC) [file pntd.0000502.s001.doc]

| **Gene** (GenBank Acc. No.) | **Primers 5’- 3’ *** | **Protein Function** |
| --- | --- | --- |
| **GFP**-control | **Fwd-** GTGCCCATCCTGGTCGAG  **Rev-** CCGTTCTTCTGCTTGTCGG | Green fluorescent protein (GFP) segment: Unspecific control. |
| **Smad1**  (gb:AF215933.1) | **Fwd-** TTGGTTGGAAACAAGGAGACG  **Rev-** TTGCTGAGTCTGTCATCGGG | Involved in cell signaling mediating cellular differentiation, proliferation and apoptosis (2). |
| **Smad2**  (gb:AF232025.1) | **Fwd-** TGACTTCAGTAGCTTATCAAGAACCTG  **Rev-** TTTGTCGGCGATAATCAGCA | Triggered by growth factor; involved in extracellular signal transmission by translocating TGF β to the nucleus. Activated-Smad2 (phosphorylated) associates with Smad4 and relocate to the nucleus, regulating genes expression. (3). |
| **PKC Receptor** (gb:AF422164.1) | **Fwd-** ATGCTACCCAGCCGGATCTT  **Rev-** GTCCGTAATGTGTTGTGCGC | Dimeric receptors activating a variety of transduction cascades. (4). |
| **Calcineurin B**  (gb:AJ276885.1) | **Fwd-** AATTTGCGTTTAAAATATACGACATGG  **Rev-** CCGGGAGAGGAGAGACCTTG | Or Phosphatase B, activated by Ca^2+^ & Calmodulin, responsible for Interleucin 2 transcriptional activation, growth stimulation, and T cell response differentiation. (5). |
| RHO1 GTPase-**RHO1**  (gb:AY158212) | **Fwd-** TTGGAGATGGTGCATGCG  **Rev-** CGGCTCGAGTAGCTGCTACG | Small GTPases, involved in a variety of cellular functions *i.e.* Cellular organization/ polarization/ migration, extracellular signal transduction, and actin cytoskeletal reorganization (6). |
| RHO2 GTPase - **RHO2** (gb:AY158214.1) | **Fwd-** TTATTCGTCGTGTACTTCATGCATTT  **Rev-** CGAGAATATAACATTTAGCCGAACAAA | Similar to RHO1 functions. |
| Protein Kinase C Beta –**PKCβ** (gb: AY337620.1) | **Fwd-** TTGGATAAGTATATTGGTGTTAATTATTTCACA  **Rev-** TTGATGATCAAGTTAACGAGCAGATA | Calcium-dependent kinase, activated upon DAG binding. Present higher transcript and protein levels in miracidia and sporocysts (4). |
| **Smad4**  (gb:AY371484.1) | **Fwd-** TTGGATCAACAGGTCGGTGA  **Rev-** TGTGCCAGGTAAAGATCCTGG | Collaborating Smad, involved in many cellular functions *i.e.* differentiation, apoptosis, gastrulation, embryonic development and cellular cycle (2). |
| High voltage-activated calcium channel β subunit 2-**Calcium Channel** (gb:AY277532.1) | **Fwd-** ATCAACTTCCAGATGCTTGTGAAC  **Rev-** CCAACAGATGTAGGATTTATGCGA | The β subunit of the calcium channel is a pore formation subunit modulator, by means, α subunit (7). |
| Homolog to Epidermal Growth Factor Receptor-SER –**SCMEG** (gb:M86399.1) | **Fwd-** AAAGCCTGTCGTGTTTGGGA  **Rev-** AATATCACAACCGGCGTGAGA | Involved in signal transduction in Schistosomes, activated by the epidermal growth factor, SMEG is important in sporocysts differentiation (8). |
| **14.3.3**  (gb:U24281.1) | **Fwd-** GGAAGCGAAATGAAAGCGG  **Rev-** GTCACTGGCCCATAACGTCA | Part of a highly conserved family of proteins and current target for vaccine development against schistosomiasis (9; 10). Interacts with numerous signaling molecules *i.e.* kinases, phosphatases and transmembrane receptors, potentially mediating cell cycle control, growth, differentiation, apoptosis and cell migration. |
| **Calpain**  (gb:M74233.1) | **Fwd-** CACTGCAGATGGAGAATTTTGG  **Rev-** TCGAATGTACTCGGAATAACAACATAT | Ca^2+^ dependent cystein protease, ubiquitously expressed in many organisms, activates PKC and involved in cytoskeletal degradation. Current target for vaccine development against schistosomiasis (11). |
| **Calreticulin**  (gb:L24159.1) | **Fwd-** AATTTCACGGCGAATCACCTTA  **Rev-** GGACGTTCCCACTCACCATC | Complex Ca^2+^ binding-protein involved in different functions *e.g.* cellular homeostasis, chaperones, extracellular lectin (12). |
| Calcium ATPase 2-**Sma2** (gb:AF074400.1) | **Fwd-** CACTTTATGCCATGGAGACTTATGTTA  **Rev-** CCTTATATTCGAACACTCTTTCATGAA | ATPase ^2+^ calcium pump isoform, actively loads the sarcoplasmic reticulum with calcium necessary to maintain cellular homeostasis, motility and signaling. Three SMAs are characterized in Schistosomes. (13; 14). |
| High voltage-activated calcium channel-**Cav2a** (gb:AF361883.1) | **Fwd-** CAGATAAAAGCAGAAGAGGAATGGA  **Rev-** AAACCGTTGGACTATCTCGTTGAT | Membrane heterodimeric proteins, subunit α is the pore formation unit (7), involved in impulse propagation in excitable cells, and intracellular calcium levels regulation. |
| Phosphoenolpyruvate Carboxikinase -**Pepck** (gb:AF120929.1) | **Fwd-** CATGTGAATTGGTTCCGTTTAAATAA  **Rev-** ATTAATGTCTACGAACAACAATGAAAG | Catalyze oxaloacetate + ATP into phosphoenolpyruvate + CO_2_+ ADP. In schistosomes PECK is mediating glyceroneogenesis (glycerol production from gluthamine precursors). PEPCK inhibition impair parasites growth demonstrating the importance of glycerol production in parasite adaptation to osmotic and energetic host environment (15) |
| Elongation Factor 1 α-**EF-1α,** (gb:Y08487.1) | **Fwd-** GTTATTGATGCACCTGGGCA  **Rev-** TTGCAAGGGAATTCTGAGTGG | GTP binding protein promoting ligation of aminoacyl-tRNAs in the ribosome, essential in the eucaryotic translational complex (16). |
| Myosin light chain- **Myosin** (gb:AF071011.1) | **Fwd-** CGAGCTTTCTTACTTAAATAACATCATGAG  **Rev-** TCTTTTACTTGGAAGGACCAGCC | Responsible for actin based motility, vesicular transport and vacuolar contraction. |
| Putative Hexamer-binding Protein-**HEXBP**  (OrganismDB:Tag623) | **Fwd-** ACGTCCCGGTCATTATGCTC  **Rev-** CATAACCATGGCACTTATAGCACTG | Zinc Finger (ZF) protein with CCHC motif binding to DNA hexamers. |
| Putative Zinc Finger DHHC domain-**DHHC**  (OrganismDB: Tag 1180) | **Fwd-** GGAAAAGATTCAATGCCCAATTC  **Rev-** GGAACAGCCAATGCTTGATGT | Highly conserved ZF proteins with cysteine rich domains, DHHC proteins are potentially involved in palmytate lipids transfer, and in *C. elegans* spermatogenesis (17). |
| **Lactate Dehydrogenase** (gb:U87629.1) | **Fwd-** CCAACAGATATCGAGCCTCGTT  **Rev-** AGGTAAGGGCGACACCCAA | Responsible for the interconversion of piruvate to lactate with concomitant conversion of NADH and NAD. Possible target for anti-schistosomotics drugs, as Pranziquantel (18) |
| SmRbx-**SmRing Box** (gb:DQ466078.1) | **Fwd-** AAAGGTCTTCAGCGCGC  **Rev-** TTATCTGAACAGGTACAACCGTAAC | Interacts with Cullin1 and potentially involved in a complex ubiquitinating proteins, directing them to degradation via 26S proteasome (19) |
| Putative **Fibrillarin** (OrganismDB: Tag 428) | **Fwd-** GCCAAAGGCAAAGAAGACTG  **Rev-** ATGGATATAACGGCGTGACC | Component of snRNP and possibly involved in pre-ribosomal RNA processing (20). |
| Glutathione S-transferase 26-**GST26** (gb:M73624.1) | **Fwd-** GTGTGGCGAAACGAAAAGTT  **Rev-** CCAACCTTGTAGAGGCCATT | Catalyzes detoxification by thiol-conjugation, reducing Gluthatione (GSH). In *S. mansoni*, possibly involved in parasite surface, neutralizing membrane damage generated by the host immune response. Potential target to vaccine development (21; 22). |
| Glutathione peroxidase-**GPx** (gb:M86510.1) | **Fwd-** CCGTGGTCACGTTTGTCTAA  **Rev-** CAAATGGCAACCAATGAACA | Protects cellular structures against oxidative damage, catalyzes H_2_O_2_ to maintaining the DNA and membrane integrity. *S. mansoni* GPX activity significantly increased when exposed to host environment, and seems to be positively correlated to the parasites antioxidant resistance (23). |
| Glutathione S-transferase 28 -**GST28** (gb:S71584.1) | **Fwd-** ATTGGCCAAAAATCAAACCA  **Rev-** TTTCCTGTCGACCCTTTCAG | Similar to GST26 functions. |
| Thioredoxin Peroxidase 2- **TPx-2** (gb:AF157561.1) | **Fwd-** CCTGCTCCTGATTTTGAAGG  **Rev-** CCAGTTCGCTGGACAAACTT | Involved in the redox balance, signaling, phosphorilation, transcription regulation and apoptosis. Reduce H_2_O_2_ in a thioredoxin-dependent manner using thioredoxin as an electron donor (24). |
| Thioredoxin peroxidase **TPX-1** (gb:AF121199.1) | **Fwd-** CCTGCACCAGAATTCAAAGG  **Rev-** CTTCACCATGCTTCTCCACA | Similar to TPX2 functions. |
| Superoxide Dismutase- **SOD** (gb:M27529.1) | **Fwd-** TTTGATCCGGCTATTGCTTC  **Rev-** TGGTACGTCCAACAAAAATCA | Metalloenzyme catalyzing the dismutation of superoxide radical to molecular oxygen and H_2_O_2_ as a defense against oxygen toxicity. Occurs in three forms: cytosolic, mitochondrial, and extracellular (25). Tested as target for vaccination resulting in 54 % worm burden reduction (26). |
| **K5** (gb:AY903301.1) | **Fwd-** CCTTCTAATAAACGGAGTCACTTTACTG  **Rev-** AACCATCGAATTGTCATCAAATCTAAT | *S. mansoni* egg secreted glycoprotein, potential target for immunodiagnostics (27). Important in transformation of miracia to sporocysts. |
| Zinc Finger 1-**SmZF1**  (gb:AF316827.1.) | **Fwd-** ACTATGGAATTTTACTTCACA  **Rev-** AGAAGTTTCGCTGGCATACTTCACAT | Binds to DNA and RNA in a specific manner, may be a *S. mansoni* transcriptional factor (28). |
| **SPO1**  (gb:AF109180.1) | **Fwd-** TCTTCCAGTAAAATGAAAGTGACG  **Rev-** TTTTCATCAATCTTTATTATTTGCTCA | Or Sm16, preferentially expressed in larval stages of schistosome parasite; it seems to play a role in the host immunoregulation mechanism (29). |

**All primers were designed with the T7 promoter sequence on its 5’ end : 5’-taatacgactcactataggg-3’**

**Table 1:**  List of genes targeted in the RNAi screening, includes specific forward and reverse primers used to amplify the 500bp templates for dsRNA synthesis. Also, protein functions were included in the context of *S. mansoni* when possible. Gb: GenBank.

| **Gene** | **Forward 5'-3'** | **5’ Position** | **Reverse 5'-3'** | **3’ Position** |
| --- | --- | --- | --- | --- |
| **Alpha-tubulin** | CAAATGGGAAATGCTTGTTG | 43 | TGAACGAGTCATCACCACCT | 148 |
| **GAPDH** | TCGTTGAGTCTACTGGAGTCTTTACG | 605 | AATATGAGCCTGAGCTTTATCAATGG | 670 |
| **Calcineurin B** | TTGCAAGGGTTATCGAGATTT | 187 | TTGGCTTCTTTCTCACCCTT | 295 |
| **PKC β** | CTTGGATGTGGTTCAGATGG | 327 | TCACGTGGTGATAAAGTAACTGG | 454 |
| **SmZF1** | ACTTCTCTCAGAAATCCAGCCT | 2 | TGGAGAGGATTATACAATCTGGTT | 98 |
| **SmRing Box** | GGCATCCCTCAGTGAGAATAA | 55 | TCCGGCAGATAGCACAATTA | 158 |
| **Myosin** | CCGTGTCCTTCTTTCTATGTTG | 2 | AGGATTCATGGAATTGTGAAAA | 100 |
| **SPO1** | CCGAGTGAAAAAGACATGGA | 50 | TGACCGTTTTATTTCGTTGC | 142 |
| **Glutathione S Transferase (GST26)** | TCAAAGGCCTTGTACAACCA | 52 | CGTCATTGCGATCATACAAA | 135 |
| **Smad1** | CCGGAAACAGTGGAACTTCT | 54 | CTTGATTGGGTTGAGTTTGG | 155 |
| **Smad2** | TTTAGCTGCCCTCCTAGCTC | 726 | TATACGCGTCAAGTGCATCA | 832 |
| **Smad4** | TGCTTCGTCTGAGTTTCGTT | 10 | AAGAGGCATAGCCTGGAGAA | 146 |
| **RHO2** | CTTCTGTCCAAATGTTCCGA | 316 | ATTCCTGACGAACTTTGGCT | 413 |
| **Elongation Factor 1 alpha** | ATGGCGATGCAGCTAACATA | 432 | TCATATCTCGAACGGCAAAG | 533 |
| **Lactate Dehydrogenase** | CCACTTGAACTTGGTGACGAT | 901 | CATCAGCAACCATGAGTCGT | 994 |
| **Thioredoxin Peroxidase (TPx-1)** | GTGAAGTGTGTCCGGTGAAC | 19 | CGGTGATCAATGAAGAACGA | 108 |
| **Thioredoxin Peroxidase (TPx-2)** | TCTTTGAGAAACATGGCGAA | 38 | GAGAGAAGCAACAGGATCAGG | 120 |
| **Glutathione S Transferase (GST-28)** | CTGGCAAGTATCCTGAGATCC | 90 | TCGTTACACCGAGCTTTCTG | 219 |
| **Glutathione Peroxidase (GPx)** | TCTCGCTATATGACGATGGC | 35 | GCAATCAGGTGCACACAAA | 113 |
| **Superoxide Dismutase (SOD)** | GATCCGGCTATTGCTTCATT | 106 | CTGCCACGCTTCCATTAAC | 202 |
| **PEPCK** | AAGTTGGTCGAATTGGGTTC | 250 | GGCACATTTGGCACAGTATC | 376 |
| **Fibrillarin** | AGAGAGGCGGGTTTAGAGGT | 131 | CTCGTGGTGTTCCTCTGTTG | 241 |
| **Calpain** | TCACTGGGTGGAAGTGCTTA | 784 | TCCAACAGAGCTGACCAGAA | 894 |
| **14.3.3** | TTCATATCGCCAAACTTGCT | 56 | CGTTCTTCGTTTCCTAAATTCC | 158 |
| **K5** | TTGCGAAGCTCAGTCTCCTA | 66 | TATTCGGACTCAACGCCATA | 187 |
| **Cav2a** | AACATCACAGGGCAGGTTTA | 346 | AAGGCACTAGCGCTTGGAT | 436 |

**Table 2:** List of primers used to quantify specific transcripts during real-time Q-PCR analysis.

**Supplemental references:**

1. Attisano L, Wrana JL (2000) Smads as transcriptional co-modulators. Curr Opin Cell Biol 12:235-243.
2. Wrana JL, Attisano L (2000) The Smad pathway. Cytokine Growth Factor Rev 11:5-13.
3. Bahia D, Andrade LF, Ludolf F, Mortara RA, Oliveira G (2006). Protein tyrosine kinases in *Schistosoma mansoni*. Mem Inst Oswaldo Cruz 1:137-143.
4. Lin X, Sikkink RA, Rusnak F, Barber DL (1999) Inhibition of calcineurin phosphatase activity by a calcineurin B homologous protein. J Biol Chem 274:36125-36131.
5. Spencer AG, Orita S, Malone CJ, Han M (2001) A RHO GTPase-mediated pathway is required during P cell migration in *Caenorhabditis elegans* PNAS 98: 13132–13137.
6. Kohn AB, Lea J, Roberts-Misterly JM, Anderson PA, Greenberg RM (2001) Structure of three high voltage-activated calcium channel alpha1 subunits from *Schistosoma mansoni*. Parasitology 123:489-497.
7. Vicogne J, Cailliau K, Tulasne D, Browaeys E, Yan YT, Fafeur V et al.. (2004) Conservation of epidermal growth factor receptor function in the human parasitic helminth *Schistosoma mansoni* J Biol Chem 279:37407-37414.
8. Mhawech P (2005) 14-3-3 proteins-an update. Cell Res 15:228-236.
9. Siles-Lucas M, Uribe N, López-Abán J, Vicente B, Orfao A, Nogal-Ruiz JJ et al. (2007) The *Schistosoma bovis* Sb14-3-3zeta recombinant protein cross-protects against *Schistosoma mansoni* in BALB/c mice. Vaccine 25:7217-7223.
10. Hota-Mitchell S, Siddiqui AA, Dekaban GA, Smith J, Tognon C, Podesta RB (1997) Protection against *Schistosoma mansoni* infection with a recombinant baculovirus-expressed subunit of calpain. Vaccine 15:1631-1640.
11. Coppolino MG, Dedhar S (2006) Calreticulin. Int J Biochem Cell Biol 30:553-558.
12. Da'dara A, Tsai MH, Tao LF, Marx KA, Shoemaker CB, Harn DA, et al. (2001) *Schistosoma mansoni*: molecular characterization of a tegumental Ca-ATPase (SMA3). Exp Parasitol. 98:215-222.
13. Talla E, de Mendonça RL, Degand I, Goffeau A, Ghislain M (1998) *Schistosoma mansoni* Ca2+-ATPase SMA2 restores viability to yeast Ca2+-ATPase-deficient strains and functions in calcineurin-mediated Ca2+ tolerance. J Biol Chem 273:27831-27840.
14. Khayath N, Mithieux G, Zitoun C, Coustau C, Vicogne J, Tielens AG, Dissous C (2006) Glyceroneogenesis: an unexpected metabolic pathway for glutamine in *Schistosoma mansoni* sporocysts. Mol Biochem Parasitol 147:145-153.
15. Tatsuka M, Mitsui H, Wada M, Nagata A, Nojima H, Okayama H (1992) Elongation factor-1 alpha gene determines susceptibility to transformation. Nature 359:333-336.
16. Gleason EJ, Lindsey WC, Kroft TL, Singson AW, L'hernault SW (2006) spe-10 encodes a DHHC-CRD zinc-finger membrane protein required for endoplasmic reticulum/Golgi membrane morphogenesis during *Caenorhabditis elegans* spermatogenesis. Genetics 172:145-158.
17. Lu G, Hu X, Peng Z, Xie H, Li Y, Wu Z, Yu X (2006) Expression and characterization of lactate dehydrogenase from *Schistosoma japonicum*. Parasitol Res 99: 593–596
18. Santos DN, Aguiar PHN, Lobo FP, Mourão MM, Tambor JHM, Valadão AF et al. (2007) *Schistosoma mansoni*: Heterologous complementation of a yeast null mutant by SmRbx, a protein similar to a RING box protein involved in ubiquitination. Experimental Parasitology 116: 440-449.
19. Amim LH, Pacheco AG, Fonseca-Costa J, Loredo CS, Rabahi MF, Melo MH et al. (2007) Role of IFN-gamma +874 T/A single nucleotide polymorphism in the tuberculosis outcome among Brazilians subjects. Mol Biol Rep. 35:563-566.
20. Bergquist R (2000) Prospects of vaccination against schistosomiasis. Scand J Infect Dis Suppl 76:60-71.
21. Gobert GN, Jones MK, Stenzel DJ. (1998) Ultrastructural analysis of the adult *Schistosoma japonicum* by lectin cytochemistry. Int J Parasitol 28:1445-1452.
22. Williams DL, Pierce RJ, Cookson E, Capron A (2000) Molecular cloning and sequencing of glutathione peroxidase from *Schistosoma mansoni*. Mol Biochem Parasitol 52:127-130.
23. Kwatia MA, Botkin DJ, Williams DL (2000). Molecular and enzymatic characterization of *Schistosoma mansoni* thioredoxin peroxidase. J Parasitol 86:908-915.
24. Simurda MC, van Keulen H, Rekosh DM, LoVerde PT (1998) *Schistosoma mansoni:* Identification and analysis of an mRNA and a gene encoding superoxide dismutase (Cu/Zn) experimental parasitology 67: 73-84.
25. Cook RM, Carvalho-Queiroz C, Wilding G, LoVerde PT (2004) Nucleic acid vaccination with *Schistosoma mansoni* antioxidant enzyme cytosolic superoxide dismutase and the structural protein filamin confers protection against the adult worm stage. Infect Immun. 72:6112-6124.
26. Hamilton JV, Chiodini PL, Fallon PG, Doenhoff MJ (1999) Periodate-sensitive immunological cross-reactivity between keyhole limpet haemocyanin (KLH) and serodiagnostic *Schistosoma mansoni* egg antigens. Parasitology 118:83-89.
27. Calzavara-Silva CE, Prosdocimi F, Abath FG, Pena SD, Franco GR (2004) Nucleic acid binding properties of SmZF1, a zinc finger protein of *Schistosoma mansoni*. Int J Parasitol 34:1211-1219.
28. Rao KV, Ramaswamy K (2000) Cloning and expression of a gene encoding Sm16, an anti-inflammatory protein from *Schistosoma mansoni*. Mol Biochem Parasitol 108:101-108.
